# Unusual Presentation of Adenoid Cystic Carcinoma of the Tongue: A Case Report and Review of the Literature

**DOI:** 10.7759/cureus.60825

**Published:** 2024-05-22

**Authors:** Archana M Sonone, Alka Hande, Swati K Patil, Aayushi Pakhale, Preethi Sharma, Sakshi Akolkar

**Affiliations:** 1 Oral Pathology and Microbiology, Sharad Pawar Dental College and Hospital, Datta Meghe Institute of Higher Education and Research (DU), Wardha, IND

**Keywords:** tubular pattern and solid pattern, cribriform pattern, malignant salivary gland neoplasm, tongue, adenoid cystic carcinomas

## Abstract

Adenoid cystic carcinoma (ACC) is a rare malignant tumor that affects the salivary glands. Its notable characteristics include aggressive local growth, infiltration of nerves (perineural invasion), a propensity to disseminate to other parts of the body (metastasize), and a high likelihood of recurrence. Here, we present the case of a 71-year-old male patient who presented with swelling on the posterior left side of his tongue, which had been causing him difficulty in chewing for the past six months. The parotid gland is frequently impacted in the head and neck area, with the tongue being comparatively uncommon. While distant metastasis is frequent, metastasis to nearby lymph nodes is not as common. However, if it does occur, it is associated with a poor prognosis and reduces the average survival age of the patient. The preferred treatment for ACC is surgical removal with wide resected margins. If it metastasizes to lymph nodes, then adjunct therapy is the treatment modality for the lesion. ACC exhibits three histopathological patterns: solid, tubular, and cribriform. The solid type is associated with a poorer prognosis compared to cribriform type, which typically has a better prognosis. This case, occurring on the tongue, is rare.

## Introduction

Malignant salivary gland tumors represent around 2-6% of the total cases of head and neck cancers (HNC) [1]. Tumors of the salivary gland are a very uncommon kind of disease with a broad variability in their histological and biological aspects, the main challenge is identifying them [2]. These tumors have modest incidence rates, several overlapping traits, and considerable variety in their morphology [3].

Adenoid cystic carcinomas (ACCs) stand as the second most prevalent type of malignant salivary gland tumor, making up roughly 5% to 10% of salivary gland neoplasms. These neoplasms, in turn, constitute approximately 2% to 4% of HNC. These lesions often occur in minor salivary glands, especially in the palate, accounting for around 31% of cases, but they can also be found in the parotid and submandibular glands [4]. It occurs at any age, but they are most often seen in women in their 5th and 6th decades of life [5]. Clinical characteristics include slow growth, distant metastases, and local recurrence, and one of the most outstanding features of this neoplasm, regardless of its site of origin, is its marked propensity to invade nerve tissue, i.e., perineural invasion. It is distinguished from other malignant salivary gland tumors by an early perineural invasion, a gradual yet aggressive clinical behavior [6].

Histopathologically, ACCs are typically infiltrative and unencapsulated. However, in rare cases, they may exhibit some differentiation. The proliferating cells are small, cuboidal, with scant cytoplasm and a round, hyperchromatic nucleus. These tumors typically lack pleomorphism, cellular atypia, and significant mitotic activity. The cells can arrange themselves in various patterns, including solid tubular and cribriform. One pattern often predominates, although multiple patterns can coexist in the same tumor. Notably, tumors with a predominantly solid pattern are considered more aggressive and have a poorer prognosis compared to those primarily tubular or cribriform [7]. Our case exemplifies the uncommon occurrence of ACC situated at the back of the tongue, showcasing a typical cribriform histopathological pattern.

## Case presentation

**Figure 1:**
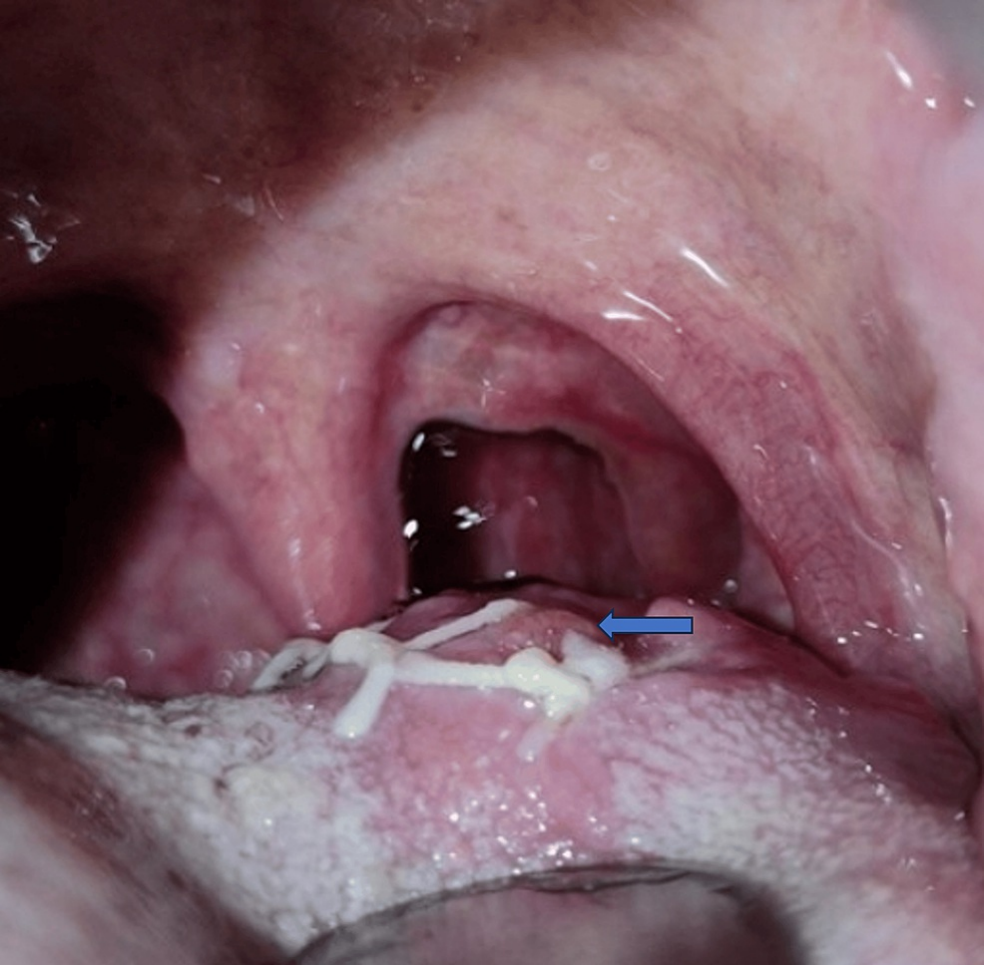
Intraoral examination shows swelling over the left posterior region of the tongue approximately of size 1.5 x 1 cm, superimposed with candidal infection.

A 71-year-old patient came up with the chief complaint of swelling in the left back region of the tongue. The patient was apparently alright six months back then he noticed swelling over the left posterior region of the tongue that was yellowish white superimposed with a candidal infection of size 1.5 x 1 cm approximately (Figure 1).

**Figure 2:**
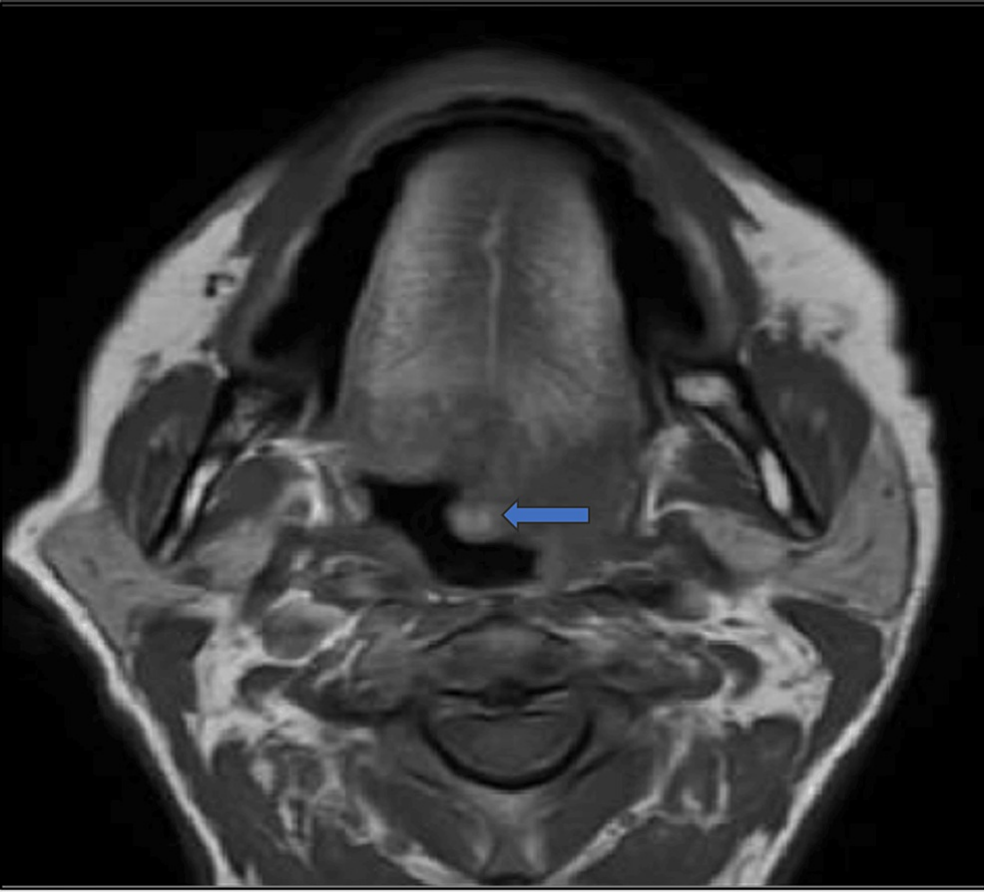
Single-intensity areas involving the left half of the tongue appearing heterogeneously hyperintense. Lesion has extensions, laterally – limited by an alveolar ridge. Medially – reaching up to midline septum. Posteriorly – Involving lingual tonsil and uvula.

There is a change in the quantity and consistency of saliva and difficulty in deglutition. The single left submandibular lymph node was palpable and not fixed to underlying structures. The MRI was done and it revealed altered single-intensity areas involving the left half of the tongue appearing heterogeneously hyperintense, involving the muscles of a tongue on the left side. The lesion has the following extensions: laterally - limited by an alveolar ridge, Medially - reaching up to midline septum, Posteriorly - involving lingual tonsil and uvula (Figure 2).

**Figure 3:**
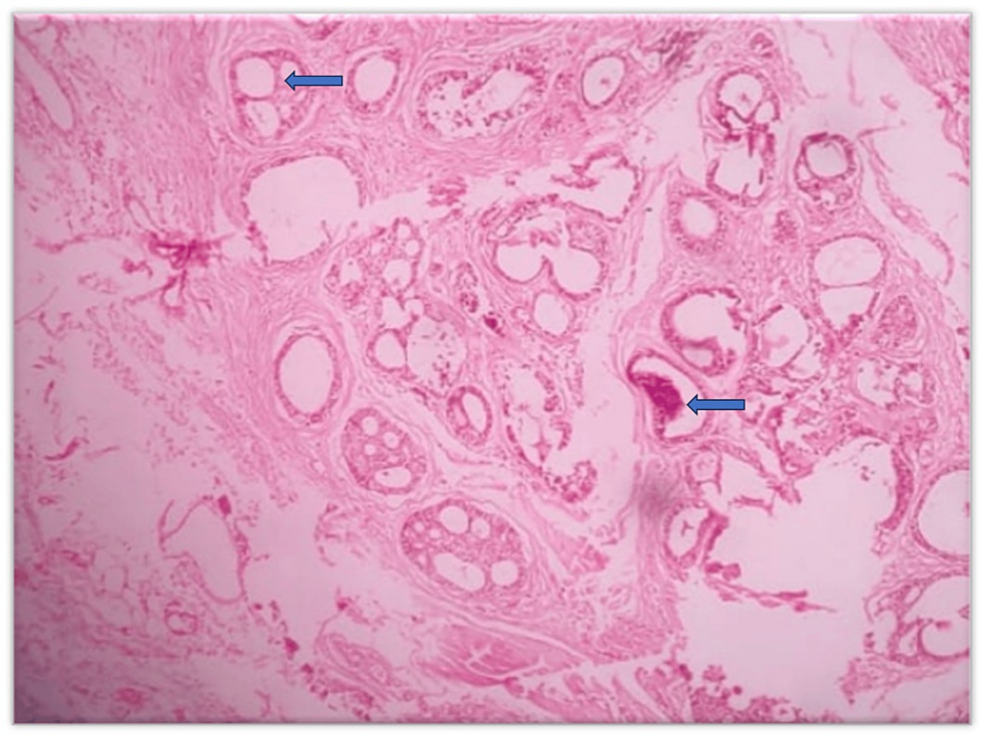
H & E stained section unveiled numerous pseudocystic spaces of varying dimensions, comprised of cuboidal cells with limited cytoplasm and oval nuclei containing eosinophilic material (100X Magnifications)

The incisional biopsy was done. The gross specimen of the incisional biopsy was roughly oval, approximately 1.4 x 1 cm in size, and color was brownish white. The histopathological analysis revealed numerous pseudo-cystic spaces of various sizes, consisting of cuboidal cells with scant cytoplasm and oval nuclei containing eosinophilic material. The pseudocystic spaces were surrounded by dense fibrous connective tissue that was well supplied with blood vessels and showed a moderate diffuse chronic inflammatory infiltration (Figure 3).

**Figure 4:**
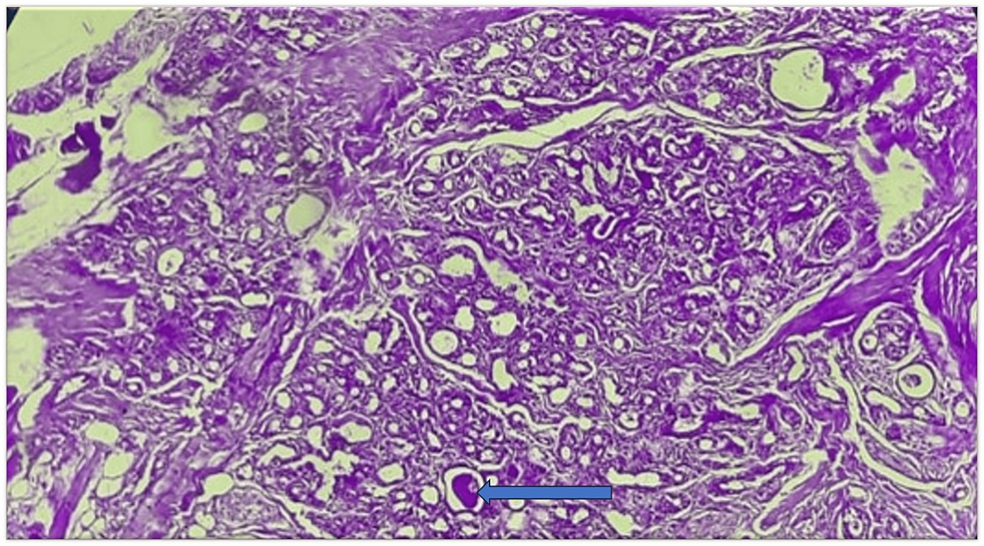
Periodic Acid-Schiff (PAS) staining - A mucinous substance showing positive PAS staining was present in the lumen (100X Magnification)

Periodic Acid-Schiff (PAS) staining of the tissue was made. The mucoid material in the cyst-like spaces of ACC showed PAS-positive staining (Figure 4).

Histopathological examination shows classical features of ACC which involves a cribriform pattern. After a histopathological confirmed diagnosis wide local excision was done under general anesthesia. After that two cycles of chemotherapy were done in which intravenous 120 mg cisplatin was given for three weeks in a four-week cycle. The patient was under observation.

## Discussion

The second most common malignant epithelial tumor of the salivary gland is adenoid cystic carcinoma (ACC). In 1859, Billroth termed it as "cylindroma" [8]. In 1953, Foote and Frazell further classified the tumor as ACC [9]. It constitutes approximately 2-4% of head and neck cancers and 5-10% of all malignant tumors originating from the salivary glands [9]. Women are primarily affected by the disease at its peak occurrence in their fifth and sixth decades of life. The submandibular area is the place with the highest frequency (30%), followed by the parotid gland. Minor salivary glands are home to 40% of cases of ACC [10].

The exact pathophysiology for tumors of the salivary gland is unknown, but various theories are there to explain the development of these tumors. In 1971, Eversole introduced the concept of the basal reserve cell, also referred to as the "progenitor cell" theory [11]. This theory advocates that basal cells within the acini act as reserve cells, generating luminal cells found in excretory and intercalated ducts. Tumors such as basal cell adenoma, canalicular adenoma, and pleomorphic adenoma are considered to align closely with this concept [11]. In 1977, Batsakis introduced the notion of the pluripotent unicellular reserve cell, proposing that basal cells found within the excretory ducts serve as reserve cells with the ability to generate all other structures within the salivary glands. Unlike Eversole's theory, this explanation does not include reserve cells from the intercalated ducts [12]. In 1981 Dardick introduced the bicellular reserve cell theory which is based on the semi-pluripotent concept which says that basal cells from both intercalated and excretory ducts play a role in forming luminal structures, but their derivatives are differentiated [13]. Basal cells from intercalated ducts produce luminal cells and acini in both intercalated and striated ducts. Whereas reserve basal cells from the excretory duct produce squamous or mucin-producing cells. Additionally, it was observed that myoepithelial cells contribute to the morphological diversity of neoplasms. Despite the widespread acceptance of the "bicellular theory of origin," there is limited empirical evidence to substantiate this notion. With the introduction of the idea of multicellular histogenesis in 1989, again he suggested that tumors could develop from the proliferation of specialized luminal and abluminal cells within mature salivary glands. Their findings showed that DNA synthesis and mitosis are both possible in duct basal cells, luminal cells, and acinar cells. Research involving the production of hyperplasia in adult rat salivary glands revealed a greater percentage of acinar cells in the S-phase of the cell cycle as compared to the S-phase cycle in intercalated ductal cells, suggesting that the salivary glands' proliferative activity goes beyond ductal basal cells [13].

The ACC demonstrates a full replication of the duct acinar unit, showcasing three unique growth patterns as: solid, tubular, and cribriform. The solid pattern emerges from an overabundance of neoplastic basal or myoepithelial cells. The tubular variant arises when luminal cells, which contribute to duct formation, are surrounded by a few cell layers of either basal cells or myoepithelial cells. In the cribriform variant, there is the presence of three types of cells maintaining the balance between the proliferative luminal cells which form ductal structures, basal cells, and myoepithelial cells [14].

The hard palate is the most frequent site for adenoid cystic carcinomas (ACCs) in minor salivary glands, making the presentation on the tongue unusual. Moran's research further supports this observation, with nine out of 38 instances of ACC occurring in the hard palate [15]. In the study by Spiro et al., accessory gland involvement was noted in 171 out of 242 cases of salivary gland adenoid cystic carcinoma (ACC). Among these cases, the palate was affected in 64 patients (26%), making it the most commonly affected site, followed by the tongue as the second most affected location [16]. Isacsson and Shear also reported neoplasm occurrences in the palate, tongue, gingiva and floor of the mouth, respectively [17].

Khan et al. noted that ACC arises from the major salivary glands in twenty-six cases out of sixty-eight cases, with the remaining cases originating from the minor salivary glands. Additionally, they reported five cases of ACC occurring in the tongue out of the total 68 cases studied [18]. In our case, the tongue is the site which is the second most common site after the hard palate. The clinical presentation of ACC on the dorsum aspect of the tongue was submucosal swelling. In this case, the patient also has swelling on the posterior part of the tongue. The sex preference for ACC is debatable, with some authors claiming that men are more prone. Eveson and Cawson found a slight prevalence of ACC cases in women (1.2:1), ranging in age from 24 to 78 years [19]. Our patient was a 71-year-old male. MRI is often preferred for imaging soft tissue lesions due to its higher accuracy compared to other modalities [20]. In this specific case, MRI was employed as the imaging technique. Histologically, adenoid cystic carcinoma (ACC) presents three distinct variants: solid, tubular, and cribriform. The solid variant typically carries the poorest prognosis due to the presence of more anaplastic cells, exhibiting more dysplastic features compared to the other two patterns. In our case, the histological pattern was cribriform, which reveals the typical Swiss cheese pattern. Soares et al. proposed a link between the histological pattern and prognosis [21]. Batsakis’ survival rate was lower in the solid design when compared to the cribriform and tubular layouts [22]. Nascimento et al. found that 17 out of 46 patients (37%) experienced local recurrence [23].

The following table represents the ACC on the tongue surface and their observations by the authors (Table 1).

**Table 1:** The table represents the ACC on the tongue surface and their observations by various authors. ACC: Adenoid cystic carcinoma; FNAC: Fine needle aspiration cytology.

SR. NO	AUTHOR	YEAR	OBSERVATION
1.	Moran et al. [15]	1960	Out of 38 cases found, they found one case on the tongue.
2.	Leafstedt et al. [24]	1971	Found 11 cases on tongue out of 56 cases of ACC.
3.	Spiro et al. [16]	1974	Twenty-six cases of adenoid cystic carcinoma were observed on the tongue out of a total of 171 cases of ACCs.
4.	Isacsson and Shear [17]	1983	Found 1% ACC on the tongue out of 200 cases of minor salivary gland neoplasms.
5.	Eveson and Cawson [19]	1985	Three cases of ACC were identified on the tongue out of a total of 336 minor salivary gland neoplasms.
6.	Nascimento et al. [23]	1986	Out of 58 cases of ACC they found eight cases on the tongue.
7.	Khan et al. [18]	2001	69 patients were observed and out of them, five ACC were on the base of the tongue.
8.	Luna-Ortiz et al. [25]	2009	Authors have done a retrospective study from 1986 to 2006, and they found eight cases of ACC, where they observed that the tongue is a rare location and early diagnosis is important for a good prognosis.
9.	Carrasco Ortiz et al. [26]	2006	Represents the case of a nineteen-year-old patient with recurrent lesions on the tongue. In the second biopsy, it was diagnosed as ACC.
10.	Baskaran et al. [20]	2012	Represents the case of a thirty-six-year-old female diagnosed with ACC on the anterior of tongue having cribriform pattern.
11.	Bansal et al. [27]	2016	The rare presentation of ACC which occurred in a 6-year-old girl child on the dorsum aspect of the tongue.
12.	Serindere et al. [28]	2017	Represents the ACC on the tongue which shows histologically tubular pattern.
13.	Garg and Gupta [29]	2019	In this case, they diagnosed the case as ACC by FNAC of metastatic lymph nodes, the lesion was on the tongue.

Patients with ACC located in the tongue often report difficulties with function, which underscores the importance of treatment strategies aimed at enhancing speech and swallowing abilities. By addressing these concerns, the overall quality of life for these patients can be significantly improved.

## Conclusions

Lesions occurring on the tongue pose unique challenges in both diagnosis and treatment, primarily due to their anatomical location and proximity to critical structures such as nerves and blood vessels. This intricate positioning complicates both the assessment of the lesion's nature and the planning of appropriate therapeutic interventions. Notably, adenoid cystic carcinoma (ACC) sites are relatively rare on the tongue compared to other locations within the oral cavity. Therefore, when ACC manifests in this particular region, it presents a somewhat atypical scenario for clinicians. In such cases, histological analysis assumes paramount importance in confirming the diagnosis of ACC. The examination of tissue samples obtained through biopsy enables pathologists to identify the characteristic features of ACC, which include the presence of cystic spaces lined by epithelial cells and the infiltrative growth pattern. Additionally, given the potential aggressiveness of ACC, assessing for the presence of distant metastases is crucial for determining the extent of the disease and guiding treatment decisions. Consequently, accurate diagnosis and comprehensive prognostic evaluation are essential for formulating an effective management strategy tailored to the individual patient. Despite the challenges posed by ACC on the tongue, advancements in diagnostic techniques and treatment modalities provide new hope for improved outcomes and better quality of life for individuals affected by this condition.
